# Guidelines, top‐notch science & social media—Jump on the bandwagon

**DOI:** 10.1002/ueg2.12203

**Published:** 2022-01-28

**Authors:** Katarzyna M. Pawlak, Lucas Wauters

**Affiliations:** ^1^ Endoscopy Unit Department of Gastroenterology Hospital of the Ministry of Interior and Administration Szczecin Poland; ^2^ Department of Gastroenterology and Hepatology University Hospitals Leuven Leuven Belgium

The beginning of a new year and 10th volume of the United European Gastroenterology (UEG) Journal marks the end of a year with publications in scientific advances and clinical strategies for the management of gastrointestinal (GI) conditions.

In 2021, the UEG and European Society for Neurogastroenterology and Motility (ESNM) consensus on functional dyspepsia (FD) and gastroparesis were published as attempts to address the lack of guidance in recognizing, diagnosing and treating these GI conditions.^1^, ^2^ Following a Delphi consensus with 41 experts from 22 European countries, a total of 87 (FD) and 89 (gastroparesis) statements were voted for agreement with evaluation of the quality of evidence.^1^, ^2^ The importance of the 36 (FD) and 25 (gastroparesis) statements reaching consensus (>80% agreement) is illustrated here for the definition, diagnosis and management of these conditions and in relation to other important publications of the past year.

While the cardinal symptoms of FD allow subdivision into epigastric pain (epigastric pain and burning) and postprandial distress syndrome (early satiation, postprandial fullness), the latter may overlap with nausea and vomiting in gastroparesis and in the presence of delayed gastric emptying.^1^, ^2^ Apart from the need of a gastroduodenoscopy for establishing a firm diagnosis of FD, endoscopy is also mandatory and useful to exclude a mechanical obstruction in gastroparesis, with a potential role for food stasis.^3^ Although dyspepsia may be managed without endoscopy in primary care patients without alarm symptoms or risk factors, the presence of *H. pylori* needs to assessed and eradicated if positive^1^ (Figure 1).

**FIGURE 1:**
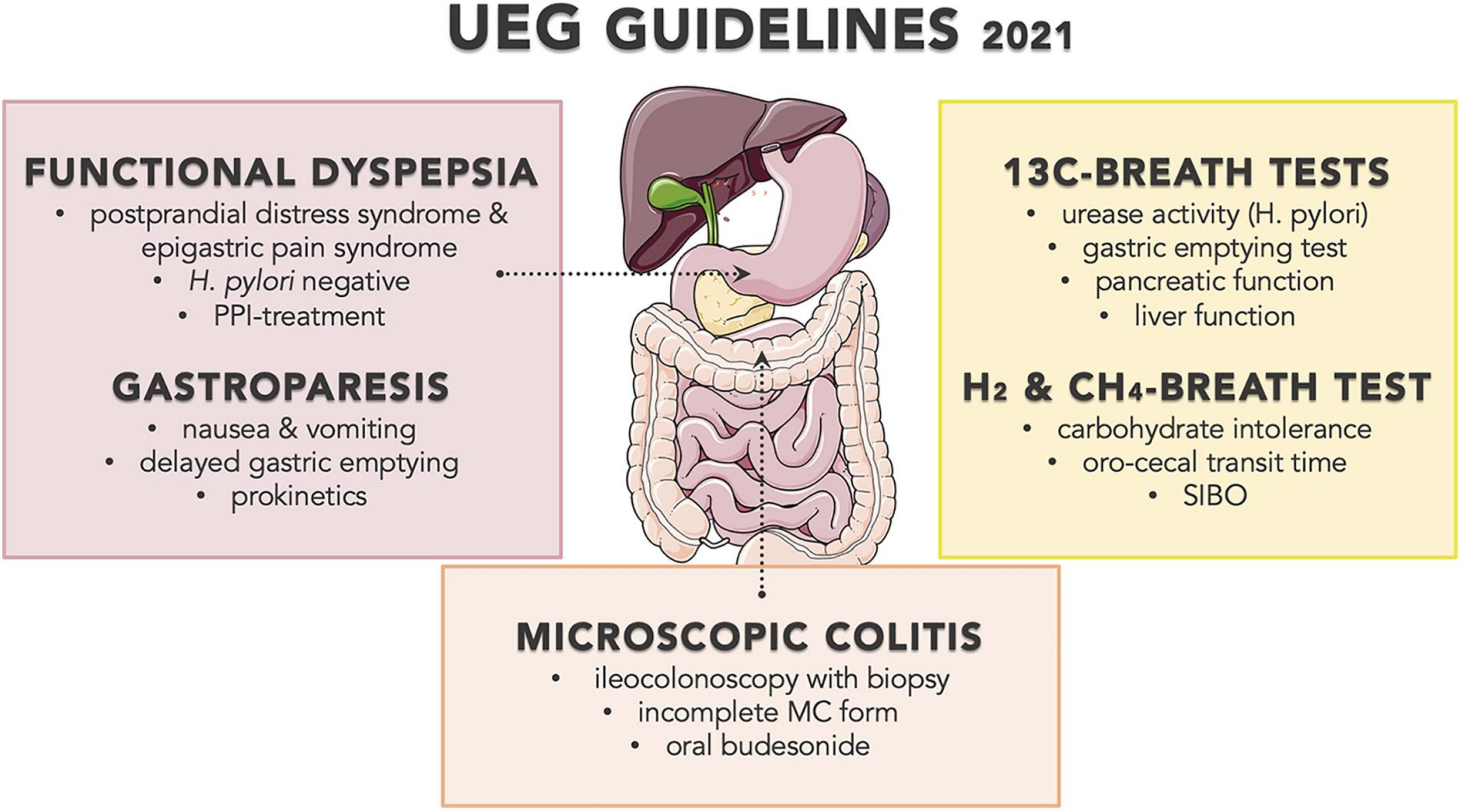
UEG Journal guidlines 2021

Guidance for the ^13^C‐urea (UBT) and gastric emptying breath tests (GEBT) is also provided by the European guideline on the use of ^13^C‐breath tests from the European Association for Gastroenterology, Endoscopy and Nutrition, ESNM and European Society for Pediatric Gastroenterology Hepatology and Nutrition, among many other tests.^4^ In this Delphi consensus with 43 experts from 18 European countries, the test‐and‐treat strategy for uninvestigated dyspepsia using the UBT received a high grade of recommendation.^4^ Also, the GEBT is an established alternative to scintigraphy, which were both endorsed in the dedicated gastroparesis‐consensus.^2^, ^4^ Of note, readers are provided with all practical measures for the performance and interpretation of these tests, including the monitoring of pancreatic exocrine and liver function^4^ (Figure 1).

In addition, the European guideline on hydrogen (H_2_) and methane (CH_4_) breath tests was recently published by a separate working group.^5^ While their use for common abdominal symptoms (e.g., bloating, flatulence, pain, and diarrhea) is increasing, the lack of standardization among centers in different countries is hampering comparisons. Besides the need for validated symptom evaluation to establish carbohydrate intolerance, the importance of confounding factors is highlighted when testing oro‐cecal transit time and suspected small bowel bacterial overgrowth (SIBO)^5^ (Figure 1). As rapid transit with early colonic fermentation of ingested sugars results in false‐positive diagnoses, this can be minimized with concomitant scintigraphy or glucose rather than lactulose.^5^ In a French monocentric and retrospective study, rotating rather than single antibiotics (quinolone or azole) were more effective for remission of SIBO using the glucose breath test, regardless of underlying disorders (including FD in up to 20%), which may be related to the use of proton pump inhibitors (PPI).^6^

Indeed, only PPI were endorsed as an effective therapy for FD, with consensus for only two classes of prokinetics in gastroparesis.^1^, ^2^ In the absence of currently approved treatments in Europe, these consensus papers identify the need for novel therapeutic approaches in FD and gastroparesis. Similar gaps have been found in the management of lower GI tract disorders, including microscopic colitis (MC) for which the UEG and European MC Group developed guidelines based on the agreement of 32 experts and researchers from 14 European countries.^7^ The consensus established the undisputed role of ileocolonoscopy as a primary diagnostic tool, including the proper biopsy performance. Importantly, experts defined the histologic criteria for incomplete MC form. However, in the monitoring process, recommendations were against the repeated histology evaluation. Finally, in the therapeutic strategy, despite the moderate level of evidence, oral budesonide was recommended as a cornerstone for the induction and maintenance treatment of both MC forms without increased risk of serious adverse events^7^ (Figure 1).

Numerous studies indicate that 25%–70% of patients on PPI have no appropriate indication for these drugs, and patients with liver cirrhosis are no exception.^8^ However, PPI increases the risk of hepatic encephalopathy and deprescribing may be a valid strategy in this situation.^9^ While all healthcare providers agree that this is an important clinical goal, it is vital to identify who has (and takes) the responsibility in starting but also stopping drugs in patients with liver cirrhosis. A review of prescriptions during multidisciplinary meetings might help.^9^

Moreover, patients with decompensated cirrhosis, presenting with complications such as ascites, variceal bleeding, and hepatic encephalopathy, have a poor survival.^10^ Patients with cirrhosis may develop sudden, acute‐on‐chronic, liver failure that results in hepatic and extra‐hepatic organ failure and is an important source of liver related mortality.^10^ Early identification of cirrhotics on the brink of decompensation may save lives and a cohort study published in the Journal found evidence that systemic inflammation drives decompensation risk.^11^ These data suggest these patients should be singled out for early assessment for liver transplant eligibility.^11^

Social media (SoMe) still remains the bone of contention in the corridors of academia. However, it seems that during the COVID‐19 pandemic, SoMe became a blessing in disguise allowing it to hold up or even improve the institutional and personal visibility, additionally remaining one of the main sources for staying up‐to‐date. Since the *@UEGJournal* appeared on Twitter in 2019, a rising interest in the UEG journal's shared content was continuously observed. A reflection of this is the increasing followers' number of the UEG Journal Twitter account, reaching almost 6000 over barely 3 years. In 2021, we put emphasis on visual graphic content presentation in SoMe, as a distinction for 29 manuscripts published in UEG journal, covering the various GI fields including inflammatory bowel diseases,^12^, ^13^ endoscopy,^14^ pancreas,^15^, ^16^ obesity,^17^ gut,^18^ GI tract physiology,^19^ and the European guidelines.^1^, ^2^ We believe that an eye‐friendly summary presentation form of published papers will beef up the UEG Journal overview and amplify the authors' effort. Another aspect of using SoMe platforms in 2021 was releasing eight episodes of the UEG Journal podcast series, giving voice to the authors, allowing for the entire route of their study process presentation—from the initial idea to the final publication of the paper.^6^, ^7^, ^20^, ^21^, ^22^, ^23^, ^24^, ^25^

The New Year is accompanied by new intentions and resolutions for further improving UEG Journal's presence in SoMe and thus continuing to provide the best access to top‐notch science.

## CONFLICT OF INTEREST

The authors declare no conflict of interest.

## Data Availability

Data sharing is not applicable to this article as no new data were created or analyzed in this study.
